# Thoracic Spinal Stability and Motion Behavior Are Affected by the Length of Posterior Instrumentation After Vertebral Body Replacement, but Not by the Surgical Approach Type: An *in vitro* Study With Entire Rib Cage Specimens

**DOI:** 10.3389/fbioe.2020.00572

**Published:** 2020-06-09

**Authors:** Christian Liebsch, Tugrul Kocak, Viktor Aleinikov, Talgat Kerimbayev, Serik Akshulakov, Jan Ulrich Jansen, Morten Vogt, Hans-Joachim Wilke

**Affiliations:** ^1^Institute of Orthopaedic Research and Biomechanics, Trauma Research Centre Ulm, Ulm University Medical Center, Ulm, Germany; ^2^Department of Orthopedics, Ulm University, Ulm, Germany; ^3^National Center for Neurosurgery, Nur-Sultan, Kazakhstan

**Keywords:** vertebral body replacement, thoracic spine, rib cage, posterior instrumentation, unilateral/bilateral approach, tumor, vertebral body fracture

## Abstract

Spinal tumors and unstable vertebral body fractures usually require surgical treatment including vertebral body replacement. Regarding primary stability, however, the best possible treatment depends on the spinal region. The purpose of this *in vitro* study was to evaluate the effects of instrumentation length and approach size on thoracic spinal stability including the entire rib cage. Six fresh frozen human thoracic spine specimens with intact rib cages (C7-L1) were loaded with pure moments of 5 Nm in flexion/extension, lateral bending, and axial rotation, while monitoring the relative motions of all spinal segments using optical motion tracking. The specimens were tested (1) in the intact condition, followed by testing after vertebral body replacement at T6 level using a unilateral approach combined with (2) long instrumentation (T4–T8) and (3) short instrumentation (T5–T7) as well as a bilateral approach combined with (4) long and (5) short instrumentation. Significant increases of the range of motion (*p* < 0.05) were found in the entire thoracic spine (T1–T12) using the bilateral approach and short instrumentation in primary flexion/extension and in secondary axial rotation during primary lateral bending compared to both conditions with long instrumentation, as well as in secondary lateral bending during primary axial rotation compared to unilateral approach and long instrumentation. Compared to the intact condition, the range of motion was significantly decreased using unilateral approach and long instrumentation in flexion extension and secondary lateral bending during primary axial rotation, as well as using bilateral approach and long instrumentation in lateral bending. On the segmental level, the range of motion was significantly increased at T4–T5 level in lateral bending using unilateral approach and short instrumentation and significantly decreased using bilateral approach and long instrumentation compared to their respective previous conditions. Regardless of the approach type, which did not affect thoracic spinal stability in the present study, short instrumentation overall shows sufficient primary stability in the mid-thoracic spine with intact rib cage, while creating considerably more instability compared to long instrumentation, potentially being of importance regarding long-term implant failure. Moreover, short instrumentation could affect adjacent segment disease due to increased motion at the upper segmental level.

## Introduction

Combined posterior fusion using pedicle screw-rod systems and anterior spinal support using vertebral body replacement implants represents the most common treatment in unstable vertebral body fractures (Lange et al., 2007; Lindtner et al., 2018). Furthermore, vertebral tumors can be treated with vertebral body replacement and posterior spinal fixation after resection of the respective vertebra (Ernstberger et al., 2005; Disch et al., 2007; Ramirez et al., 2010; Trobisch and Verma, 2015). In rare cases, vertebral body replacement with subsequent posterior fusion is also performed in the treatment of degenerative stenosis, strong deformity, or severe infection (Arts and Peul, 2008). Overall, this surgical procedure was shown to ensure adequate primary stability in the thoracolumbar spine (Vahldiek and Panjabi, 1998; Wilke et al., 2001; Pflugmacher et al., 2004).

Previous investigations showed that most vertebral body fractures occur in the thoracolumbar region, more specifically at the T12 and L1 levels (Magerl et al., 1994; Watt et al., 2015). Although vertebral body fractures were overall more often found in the lumbar spine within these studies, nearly the same number of fractures was detected in the thoracic spine (Magerl et al., 1994; Watt et al., 2015). Fracture rate per vertebra is therefore lower in the thoracic spine considering the higher number of vertebral bodies compared to the lumbar spine, which could be explained by reduced loads, the specific morphology of the spine, or the stabilizing effect of the rib cage. Nevertheless, thoracic vertebral body fractures represent a major clinical issue. Moreover, a peak of the vertebral body fracture distribution was found at the mid-thoracic spine, specifically at the T6 and T7 levels (Magerl et al., 1994; Watt et al., 2015), which also represent the apex of the thoracic spine in the sagittal plane. This leads to the assumption that thoracic kyphosis and thus the higher distance of the mid-thoracic spine from the center of mass line of the body causes higher loads in its anterior vertebral section. Consequently, this presents a higher risk of vertebral body fractures in case of high flexion moments or excessive compression forces. Spinal tumors, in contrast, generally affect the entire spinal complex. In elderly patients, about 50% of all cases of cancer are related to spinal structures (Aebi, 2003). Surgical treatment, involving vertebral body replacement, becomes necessary when spinal stability is not assured anymore (Aebi, 2003). While the extent of resection may vary, specific portions of the affected vertebra are resected in most cases (Aebi, 2003; Ernstberger et al., 2005; Trobisch and Verma, 2015). However, surgical treatment can also include resection of entire vertebrae (Stener, 1989). This usually requires a bilateral surgical approach, which involves the resection of both costotransverse and costovertebral joints of a thoracic vertebra, in contrast to a unilateral approach, which is usually performed in the treatment of small and well-defined vertebral tumors or vertebral body fractures.

The stability and motion behavior of combined vertebral body replacement and posterior spinal fusion in terms of the primary and secondary ranges of motion have mainly been investigated on the lumbar spine in the past. However, due to the stabilizing effect of the rib cage and a diverging spinal morphology, the optimum treatment for the thoracic spine may differ from that for the lumbar spine. The purpose of this *in vitro* study therefore was to evaluate the effects of (1) the length of instrumentation (one level above/one level below vs. two levels above/two levels below) and (2) the type of surgical approach (unilateral vs. bilateral approach) on thoracic spinal stability (defined as primary ranges of motion) and motion behavior (defined as secondary ranges of motion) when performing vertebral body replacement combined with posterior fusion and considering the rib cage.

## Materials and Methods

### Specimens

Six fresh frozen human thoracic spine specimens (C7-L1), including the intact rib cage, were gathered for biomechanical testing. Four of the six specimens originated from male and two from female donors, together exhibiting a mean age of 81 ± 13 years, ranging from 63 to 99 years, as well as a mean bone mineral density of 38 ± 35 mgHA/cm^3^, ranging from 2 to 96 mgHA/cm^3^ (Table 1). Prior to preparation, the specimens were screened for signs of severe deformity, degeneration, and previous vertebral fractures using computer tomography (Siemens Somatom Definition AS, Siemens Healthcare, Erlangen, Germany), which also served for the determination of the mineral content of the vertebral bodies, calculated from three separate measurements in the lower three thoracic spinal levels. During preparation, fat and muscle tissue was removed except for the intercostal muscles, leaving intact all bony, cartilaginous, and ligamentous structures. For biomechanical testing, the C7 and L1 vertebrae of the specimens were potted in PMMA (Technovit 3040, Heraeus Kulzer, Wehrheim, Germany) to half of their height and parallel to their endplates, serving as contact areas for the fixation of flanges on which the respective moments were applied. To enhance the stability of the embedding, screws were driven into the vertebrae prior to potting. The specimens were stored at −20°C and thawed for about 12 h at 5°C prior to preparation and testing, which were kept below 20 h in total. To prevent disintegration processes, all specimens were periodically moistened during testing with 0.9% saline solution.

**TABLE 1:** Donor-specific data regarding age and bone quality for the specimens used in the present study.

**Specimen no.**	**Age range (years)**	**BMD (mgHA/cm^3^)**
1	60–65	96–100
2	96–100	56–60
3	96–100	6–10
4	80–85	0–5
5	90–95	56–60
6	70–75	0–5
Mean ± SD	81 ± 13	38 ± 35

*BMD, bone mineral density, SD, standard deviation.*

### Experimental Setup

**FIGURE 1:**
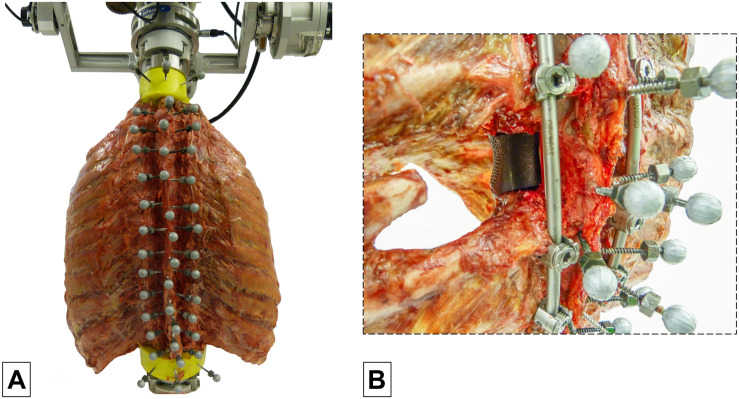
Illustration of the test setup showing a thoracic spine and rib cage specimen with three reflective markers per vertebra (C7-L1) in the spine tester **(A)** and an example of a specimen with vertebral body replacement implant at T6 level using a unilateral surgical approach stabilized by long posterior instrumentation from T4 to T8 **(B)**.

The specimens were quasi-statically loaded with pure moments of 5 Nm in the primary motion planes flexion/extension, lateral bending, and axial rotation using a well-established spine tester (Wilke et al., 1994). The moments were applied displacement-controlled with an angular velocity of 1°/s for 3.5 loading cycles to reduce viscoelastic effects, following widely used recommendations for *in vitro* testing of spinal implants (Wilke et al., 1998b). Simultaneously with load application and triggered by a voltage signal of the stepper motors, optical motion tracking of all vertebrae was performed in order to monitor the relative motions of all functional spinal units corresponding to previous *in vitro* studies on the thoracic spine with intact rib cage (Liebsch et al., 2017a, b, 2018). Therefore, each vertebra was equipped with three reflective markers (Figure 1A), which were individually captured by at least three of twelve infrared cameras (Vicon MX13, Vicon Motion Systems Ltd., Oxford, United Kingdom) surrounding the dorsal section of the specimen.

### Testing Procedure

**FIGURE 2:**
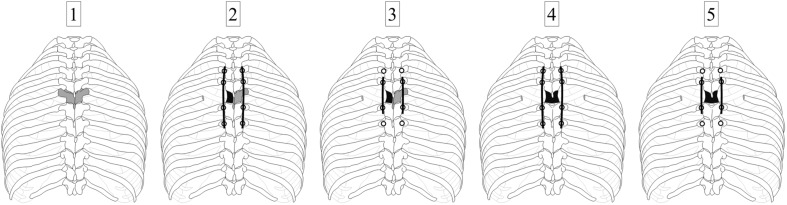
Illustration of the testing sequence: Specimens were tested **(1)** in the intact condition, **(2)** after implantation of a donor-specific 3D printed titanium vertebral body replacement at T6 level (depicted in gray) using a unilateral surgical approach and long (T4–T8) posterior instrumentation, **(3)** in the same condition but using short (T5–T7) posterior instrumentation, **(4)** after performing a bilateral surgical approach stabilized by long (T4–T8) posterior instrumentation, and **(5)** in the same condition but using short (T5–T7) posterior instrumentation.

**FIGURE 3:**
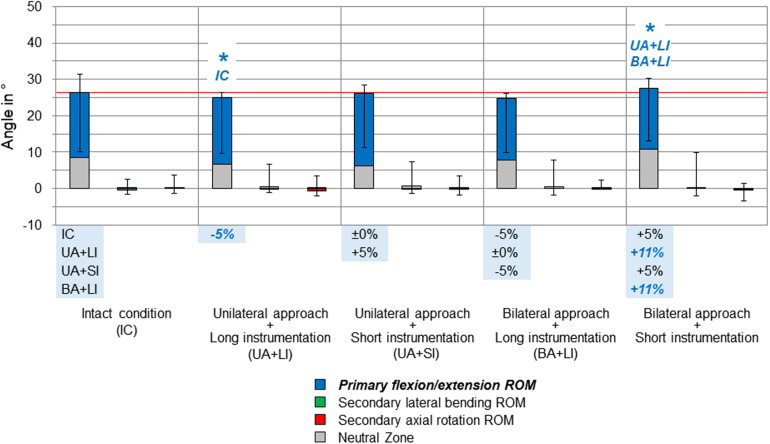
Results for primary flexion/extension movement of the entire thoracic spine (T1–T12), illustrated as medians with ranges, respectively. Significant differences (*p* < 0.05) are labeled with an asterisk as well as the respective condition to which the ROM median value was significant. Relative ROM changes between the single conditions are given at the bottom of the diagram for primary flexion/extension. The red horizontal line indicates the median value of the intact condition.

**FIGURE 4:**
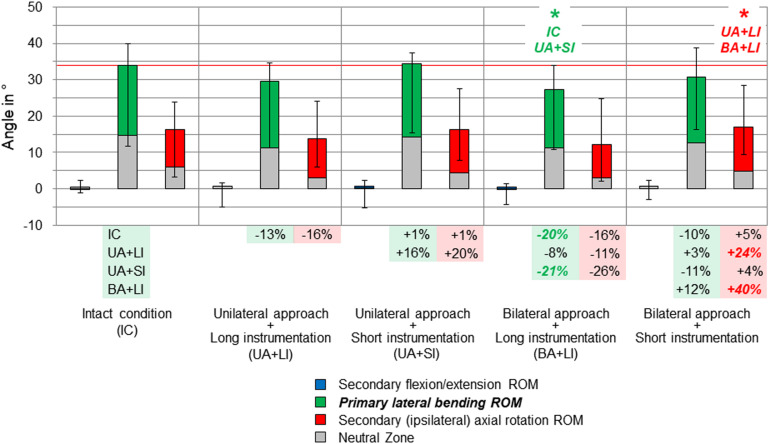
Results for primary lateral bending movement of the entire thoracic spine (T1–T12), illustrated as medians with ranges, respectively. Significant differences (*p* < 0.05) are labeled with an asterisk as well as the respective condition to which the ROM median value was significant. Relative ROM changes between the single conditions are given at the bottom of the diagram for primary lateral bending and secondary axial rotation. The red horizontal line indicates the median value of the intact condition.

**FIGURE 5:**
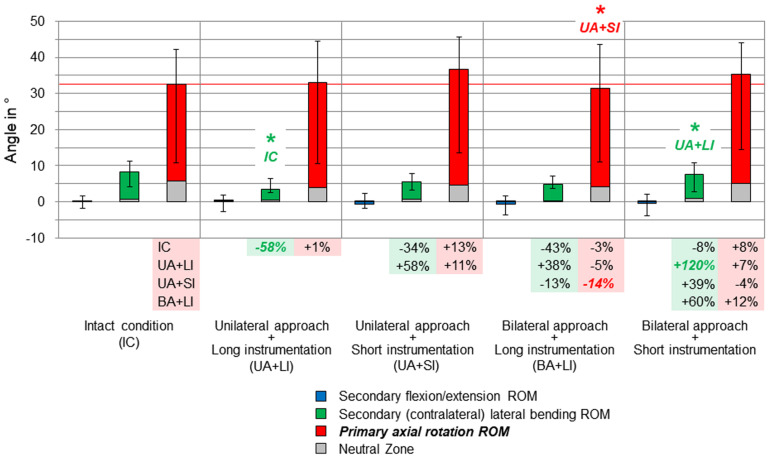
Results for primary axial rotation movement of the entire thoracic spine (T1–T12), illustrated as medians with ranges, respectively. Significant differences (*p* < 0.05) are labeled with an asterisk as well as the respective condition to which the ROM median value was significant. Relative ROM changes between the single conditions are given at the bottom of the diagram for primary axial rotation and secondary lateral bending. The red horizontal line indicates the median value of the intact condition.

The six specimens were tested stepwise in five different configurations. Surgical procedures to create these configurations were performed by two experienced spine surgeons (TKo, VA). In the first step, the specimens were loaded in the intact condition without surgical approach or posterior instrumentation (Figures 1A, 2-1). In the second step, testing was performed after surgical removal of the vertebral body of the T6 vertebra using a unilateral approach, insertion of a vertebral body replacement implant, and posterior fixation using pedicle screw-rod instrumentation from T4 to T8 (Figures 1B, 2-2), in the following referred to as long instrumentation. The unilateral approach included the removal of the adjacent intervertebral discs, the sixth left rib head, and the stabilizing ligaments of the left costovertebral and costotransverse joints, following previous descriptions (Trobisch and Verma, 2015). First, the left rib was cut about 50 mm from the left costotransverse joint using an oscillating saw (OR-SY-518.01, Synthes, Zuchwil, Switzerland). The stabilizing ligaments of the rib-vertebra joint were then cut through using a scalpel in order to remove the rib head. Using bone rongeur, chisel, and scalpel, the vertebral body of T6 and the adjacent intervertebral discs were stepwise removed, followed by implant insertion. Titanium vertebral body replacement implants were used, which were generated using specimen-specific 3D printing (JV Ghalam LLP, Nur-Sultan, Kazakhstan) from CT data acquired prior to preparation. Posterior instrumentation was realized using standard pedicle screw-rod constructs for the thoracic spine (5.0 × 40 mm pedicle screws for T4 and T5, 5.0 × 45 mm pedicle screws for T7 and T8, as well as Ø5.5 mm rods). In the third step, testing was conducted after replacing the long rods by shorter rods, simulating posterior instrumentation from T5 to T7, hereafter referred to as short instrumentation (Figure 2-3). Prior to the fourth testing step, the entire T6 vertebra was removed simulating a bilateral surgical approach including the removal of all bony vertebral structures and all stabilizing ligaments surrounding the T6 vertebra using bone rongeur and scalpel, as well as the sixth right rib head using an oscillating saw to cut the right rib about 50 mm from the right costotransverse joint. In the fourth step, the specimens were again tested using long instrumentation (Figure 2-4). In the fifth and last testing step, short instrumentation was again used (Figure 2-5).

### Data Evaluation and Statistics

Ranges of motion and neutral zones of the entire thoracic spine (T1–T12) as well as the two adjacent levels above and below the vertebral body replacement (T3–T4, T4–T5, T7–T8, T8–T9) were evaluated from the hysteresis curves of the third full loading cycles in all primary and secondary motion planes using Matlab 2017 (MathWorks Inc., Natick, MA, United States) and post-processed using Excel 2016 (Microsoft Corp., Redmond, WA, United States). All primary (in-plane) and secondary (out-of-plane/coupled) ranges of motion were grouped and statistically analyzed for significant differences using Friedman ANOVA with Bonferroni correction and a significance level of 0.05. Statistical analysis was performed using SPSS 24 (IBM Corp., Armonk, NY, United States). Grouped data were represented and visualized as median values with respective value ranges, while negative values characterized inverse motions, for instance, extension during primary flexion and vice versa.

### Ethics, Funding, and Conflicts of Interest

The use of human specimens in the present *in vitro* study was approved by the ethical committee board of the University of Ulm, Germany, in April 2018 (No. 487/17). The specimens were acquired from the anatomy department of the University Hospital Aachen, Germany, in November 2018, which declared that written informed consent of the donors was obtained prior to decease. The study was funded by the German Research Foundation (DFG, WI1352/20-2) and the Kazakhstan Ministry of Public Health (32-18-280/129). The authors declare to have no potential conflicts of interest.

## Results

Compared to the intact condition, no significant increases of the primary and secondary ranges of motion of the entire thoracic spine (T1–T12) were found for all testing steps (Figures 3–5). In primary flexion/extension, the range of motion significantly decreased by 5% after applying the unilateral approach and long instrumentation compared to the intact state, while it was significantly increased by 11% after performing the bilateral approach and short instrumentation compared to both conditions with long instrumentation and either uni- or bilateral approach type (Figure 3). No distinct changes were seen in the coupled motion values of both secondary lateral bending and axial rotation, which were overall hardly present in primary flexion/extension.

Considerable effects of the instrumentation length were found in both primary lateral bending (Figure 4) and primary axial rotation (Figure 5) regarding the range of motion of the entire thoracic spine. In primary lateral bending, the range of motion was significantly reduced after installing the long instrumentation in the bilateral approach compared to both the intact condition (−20%) and the condition in which short instrumentation and the unilateral approach type were used (−21%). In contrast, the secondary ipsilateral axial rotation, which accounted for about half of the primary lateral bending range of motion in the intact condition, was significantly increased in the condition of bilateral approach and short instrumentation compared to both conditions using long instrumentation (+24% combined with unilateral approach, +40% combined with bilateral approach, Figure 4). In primary axial rotation, long instrumentation led to a significant decrease of the range of motion regarding the secondary contralateral lateral bending with unilateral approach compared to the intact condition (−58%). In contrast, a significant increase of the secondary contralateral lateral bending range of motion was detected in the condition with bilateral approach and short instrumentation compared to the condition with unilateral approach and long instrumentation (+120%, Figure 5). Regarding primary axial rotation, the range of motion was solely decreased in the condition with bilateral approach type and long instrumentation relative to the condition with unilateral approach and short instrumentation (−14%). In both primary lateral bending and primary axial rotation, secondary flexion/extension did not alter throughout all testing steps while generally being relatively low.

**FIGURE 6:**
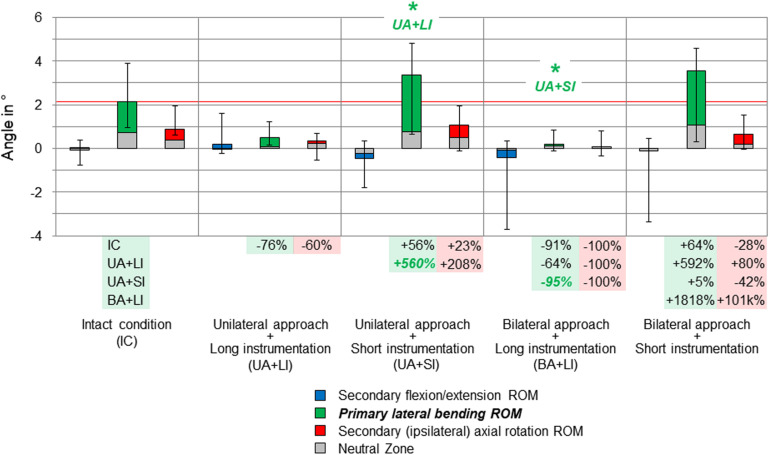
Results for primary lateral bending movement of the motion segment T4–T5, illustrated as medians with ranges, respectively. Significant differences (*p* < 0.05) are labeled with an asterisk as well as the respective condition to which the ROM median value was significant. Relative ROM changes between the single conditions are given at the bottom of the diagram for primary lateral bending and secondary axial rotation. The red horizontal line indicates the median value of the intact condition.

On the segmental level, no significant range of motion value changes were found, neither in primary nor in secondary motion planes, except for the primary lateral bending range of motion at the T4–T5 level (Figure 6). Here, unilateral approach and short instrumentation led to a significant increase of the range of motion compared to the condition with the same approach type and long instrumentation (+560%). When applying long instrumentation, the primary lateral bending range of motion significantly decreased with combined bilateral approach compared to the condition using short instrumentation and the unilateral approach type (−95%). In all motion planes of the levels T3–T4, T7–T8, and T8–T9, as well as in flexion/extension and axial rotation of the level T4–T5, the differences between the tested groups were overall low and non-significant, while exhibiting larger variation ranges but generally similar trends compared with the results for the entire thoracic spine (T1–T12).

All results are given in summarized form in the Supplementary Material files attached to this article.

## Discussion

Vertebral body replacement combined with posterior instrumentation using pedicle screw-rod constructs is widely used in the surgical treatment of spinal diseases such as unstable vertebral body fractures, spinal tumors, and other pathologies. Regarding primary stability, however, the length of instrumentation and the type of surgical approach depends on several factors, such as the spinal morphology or the effect of stabilizing ligamentous and bony structures. Since these parameters were mainly studied in the lumbar spine in the past, the present biomechanical study aimed to evaluate the effects of long and short instrumentation as well as the effects of uni- and bilateral surgical approaches on the primary stability of the thoracic spine, including all rib cage structures to represent a more physiological condition.

The findings of the present study generally revealed that thoracic spinal stability is maintained after vertebral body replacement, independent of instrumentation length or surgical approach type, when compared to the intact condition where no vertebral body replacement implant and no posterior instrumentation were used. However, short instrumentation (one level above/one level below) was found to create distinctly more range of motion compared to long instrumentation (two levels above/two levels below), especially in primary flexion/extension using the bilateral approach type. Furthermore, short instrumentation altered the coupled motion characteristics of the entire thoracic spine (T1–T12) in both primary lateral bending and primary axial rotation, leading to increased secondary axial rotation and secondary lateral bending, respectively, when using the bilateral approach type. These findings might indicate that short instrumentation more likely induces long-term implant failure or pedicle screw loosening, assuming that higher flexibility is related to higher stresses in the rods and the vertebral bone of the adjacent segments. Moreover, short instrumentation led to a significant range of motion increase at the segmental level T4–T5 in primary lateral bending using the unilateral approach type compared to long instrumentation, indicating a higher risk of adjacent segment degeneration when hypothesizing that increased range of motion in the respective motion segment is a primary risk factor for adjacent segment disease. Furthermore, variation ranges were detected to be larger after posterior instrumentation compared to the intact condition at all adjacent segmental levels and in all motion planes, indicating varying effects of the posterior instrumentation regarding level and motion direction. Regarding the surgical approach type, no clear indications were found that the bilateral surgical approach causes higher instability compared to the unilateral approach when considering comparisons between the long and short instrumentation groups, respectively. Combined vertebral body replacement and posterior fusion therefore seems to compensate the destabilization induced by the removal of all ligamentous and bony structures due to the surgical approach.

Surgical procedures were generally found to destabilize the thoracic spine in previous biomechanical studies, since it was shown that resections of spinal structures, costotransverse and costovertebral joint ligamentous structures, as well as anterior rib cage structures significantly decrease the stability of the respective thoracic spinal motion segments (Oda et al., 2002; Liebsch et al., 2017a). A previous study by Perry et al. (2014) detected a significant destabilization of the thoracic spine in all motion planes after simulating a burst fracture of the vertebral body at T9 level, which could be fully restored by sole posterior instrumentation, sole vertebral body augmentation, and combined posterior instrumentation and vertebral body augmentation, respectively. Moreover, Perry et al. did not find any differences between long and short posterior instrumentation. However, the effects of vertebral body replacement, as used for severely unstable fractures, as well as a resection of vertebral structures, as performed in surgical tumor therapy, were not investigated in their study. Further studies did not detect any destabilizing effects after surgical decompression at T4–T5 level (Healy et al., 2014) and T8–T9 level (Lubelski et al., 2014), whereas following posterior instrumentation two levels above and three levels below significantly decreased the thoracic spinal range of motion. In sum, all these findings indicate that resection of posterior thoracic spinal structures can be compensated by the stabilizing effect of the rib cage, whereas resection of the thoracic vertebral body leads to a severe loss of stability, which has to be treated at least with posterior fusion. Furthermore, the results of the present study suggest that vertebral body resection should rather be supported with long posterior instrumentation to maintain the stability in the primary, but also in the secondary motion planes, which were additionally evaluated because of the strong coupling relationship between lateral bending and axial rotation found in previous *in vitro* studies (Liebsch et al., 2017a, 2018), potentially affecting long-term three-dimensional spinal deformity.

For a correct interpretation of the findings in the present *in vitro* study, several methodological limitations have to be considered. Due to the quasi-static test setup, the present study solely investigated the effects of instrumentation length and surgical approach type on the primary stability of the thoracic spine. For analyses of long-term effects, such as implant failure or sintering, pedicle screw loosening, or tissue damage, a dynamic test setup including high load cycle numbers would have been required. However, dynamic test setups using entire rib cage specimens would have entailed further limitations, such as the potential risk of specimen disintegration due to longer testing periods and viscoelastic artifacts following high loading velocities (Wilke et al., 1998a). Moreover, a clinical study on thoracolumbar burst fractures showed that long-term postsurgical deformity is rather related to age and morphological properties, such as anterior vertebral height or the vertebral body wedge angle, than to the instrumentation length (Chen et al., 2016). Another limitation is the absence of a testing step simulating a vertebral body fracture or a vertebral tumor in order to get information about the stabilizing effect of surgical treatment using vertebral body replacement and posterior spinal fixation. Preliminary tests, including trials without vertebral body, however, exhibited enormous ranges of motion at the mid-thoracic levels, suggesting the risk of tissue damage and potentially affecting the subsequent testing steps due to premature specimen failure. Moreover, increasing the number of testing steps would have further increased the potential effect of the testing order on the results. In this study, the testing order was not randomized due to the low sample size and the high number of testing steps as well as due to the resection of tissue between the single steps, making a randomization generally impossible. Furthermore, the testing order could not be randomized, because the long rod configuration had to be tested prior to the short rod configuration in order to further prevent specimen damage between the single steps and the priority was set to the reproducibility of the testing order. However, since quasi-physiological loads were applied in this study and the recommended testing period was not exceeded (Wilke et al., 1998b), it can be assumed that the testing order should not have essentially affected the outcome of the present study. Finally, statistical differences between the single groups could have been affected by the Bonferroni correction, which was used to take cumulated α errors between the single groups into account, meaning that differences between certain groups could have been significant if the number of groups would have been reduced. However, the findings of this study were not affected by this method, since the Bonferroni correction generally reduces the significance levels. Moreover, the study design had to include all five groups in order to draw clear conclusions about the effect of both instrumentation length and surgical approach. Future studies should therefore further investigate the role of posterior instrumentation in thoracic spinal stabilization, especially with regard to upper and lower thoracic spinal levels and multiple level treatment.

## Conclusion

This study showed that long instrumentation (two levels above/two levels below) may be generally recommended after vertebral body replacement in the thoracic spine to avoid the risks of implant failure, pedicle screw loosening, or adjacent segment disease. Short instrumentation (one level above/one level below), however, also ensures reasonable primary stability and thus is an acceptable alternative in individual cases, for instance, when the extent of surgical approach has to be reduced for medical reasons. The bilateral surgical approach type, as used for the treatment of vertebral tumors, represents a secure surgical procedure from a biomechanical point of view when combined with vertebral body replacement implant and posterior fusion. Nevertheless, the bilateral surgical approach entails a major destabilizing surgical intervention which should be treated with at least long posterior instrumentation.

## Data Availability Statement

All datasets generated for this study are included in the article/Supplementary Material.

## Ethics Statement

The use of human specimens in the present *in vitro* study was approved by the ethical committee board of the University of Ulm, Germany, in April 2018 (No. 487/17). The specimens were acquired from the anatomy department of the University Hospital Aachen, Germany, in November 2018, which declared that written informed consent of the donors was obtained prior to decease.

## Author Contributions

CL contributed to study design, experimental testing, data analysis, and manuscript preparation. TKo contributed to experimental testing, discussion, and manuscript review. VA contributed to funding acquisition, experimental testing, discussion, and manuscript review. TKe contributed to funding acquisition and manuscript review. SA contributed to funding acquisition and manuscript review. JJ contributed to experimental testing, data analysis, and manuscript review. MV contributed to experimental testing, data analysis, and manuscript review. H-JW contributed to funding acquisition, discussion, and manuscript review.

## Conflict of Interest

The authors declare that the research was conducted in the absence of any commercial or financial relationships that could be construed as a potential conflict of interest.
