# Supplementary figures and images for: Thoracic Spinal Stability and Motion Behavior Are Affected by the Length of Posterior Instrumentation After Vertebral Body Replacement, but Not by the Surgical Approach Type: An in vitro Study With Entire Rib Cage Specimens
Source: Front Bioeng Biotechnol. 2020 Jun 9;8:572. doi: 10.3389/fbioe.2020.00572 (PMC7295896; doi:10.3389/fbioe.2020.00572)

# T1-T12

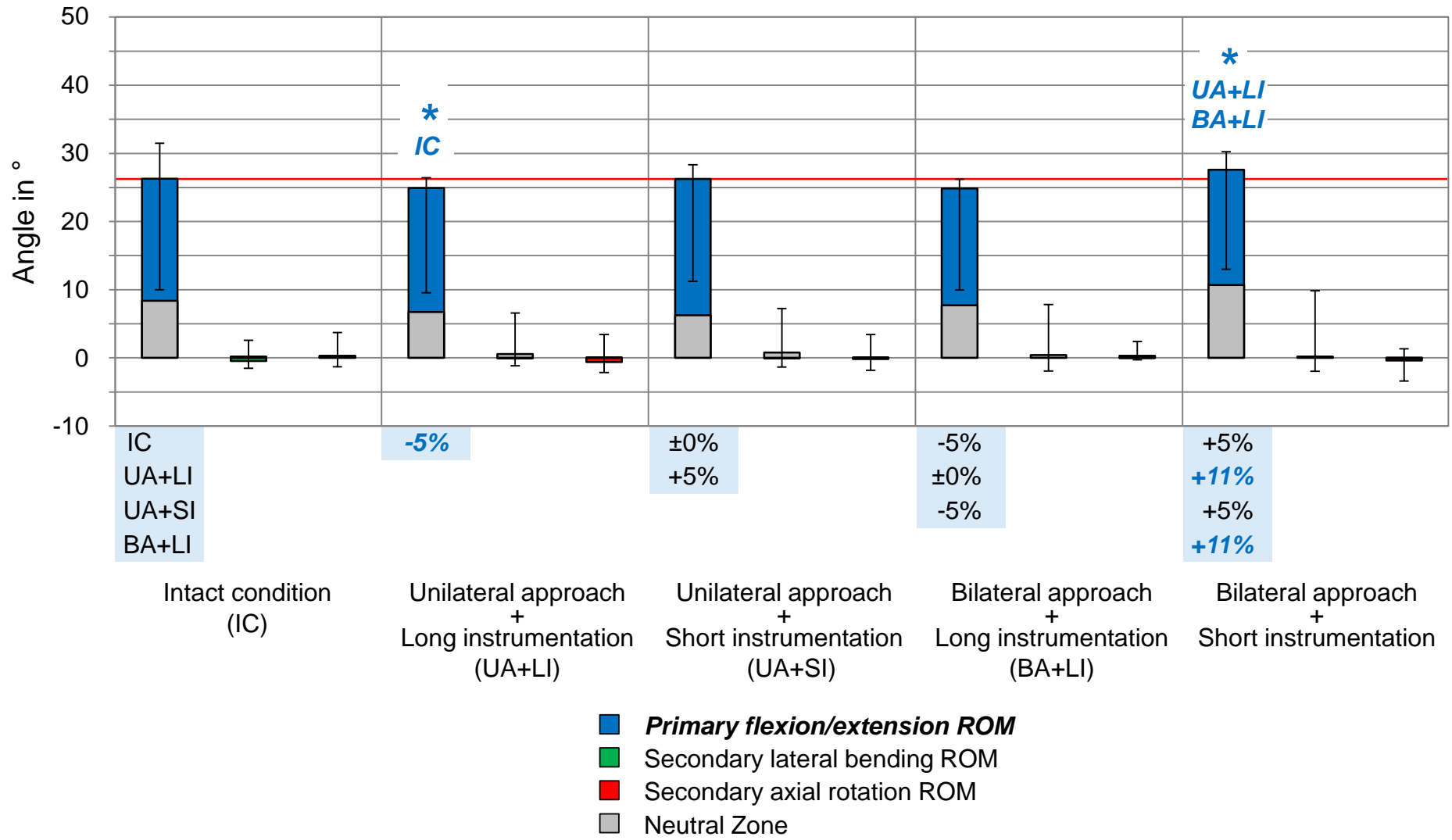

# T1-T12

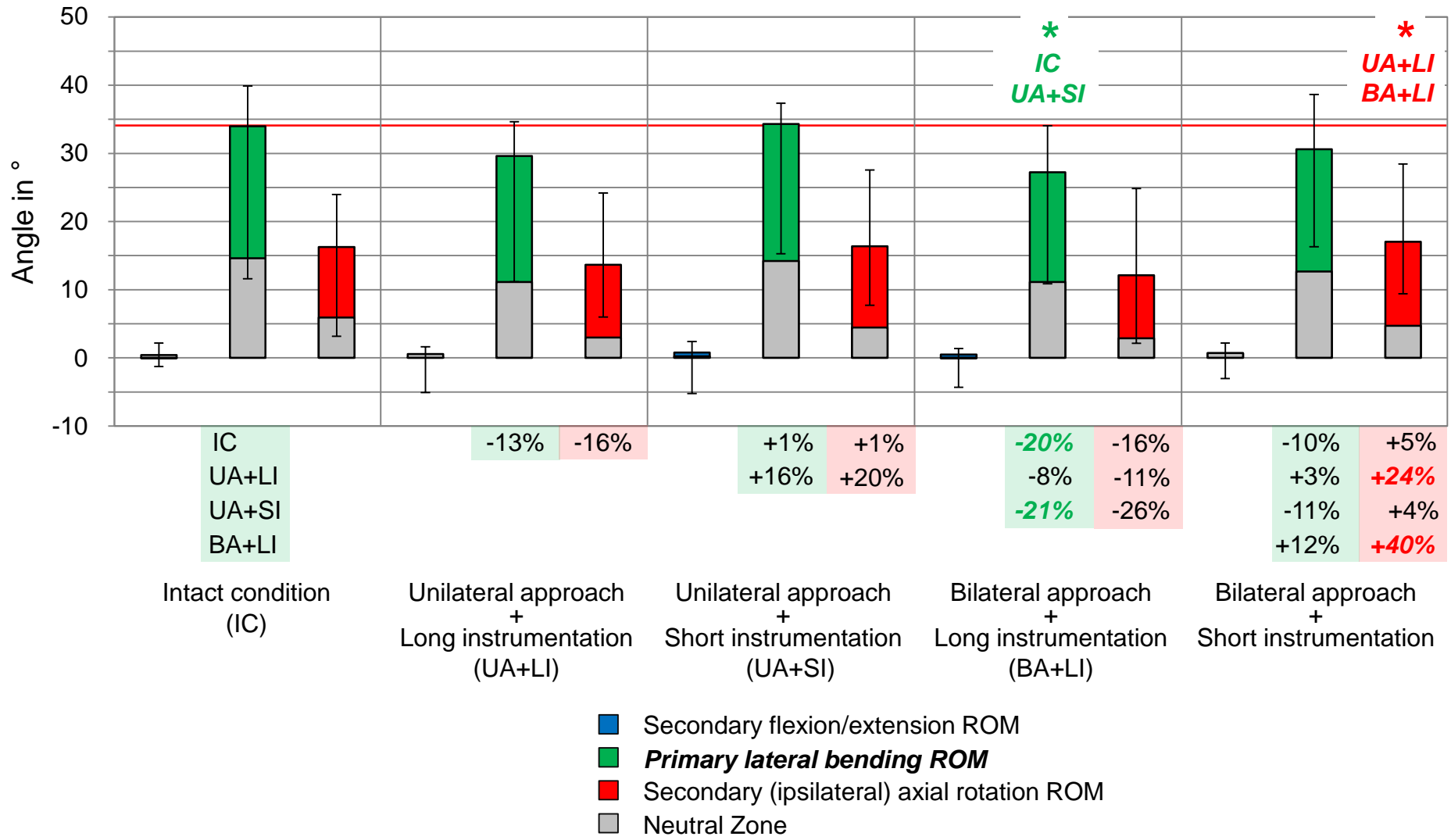

# T1-T12

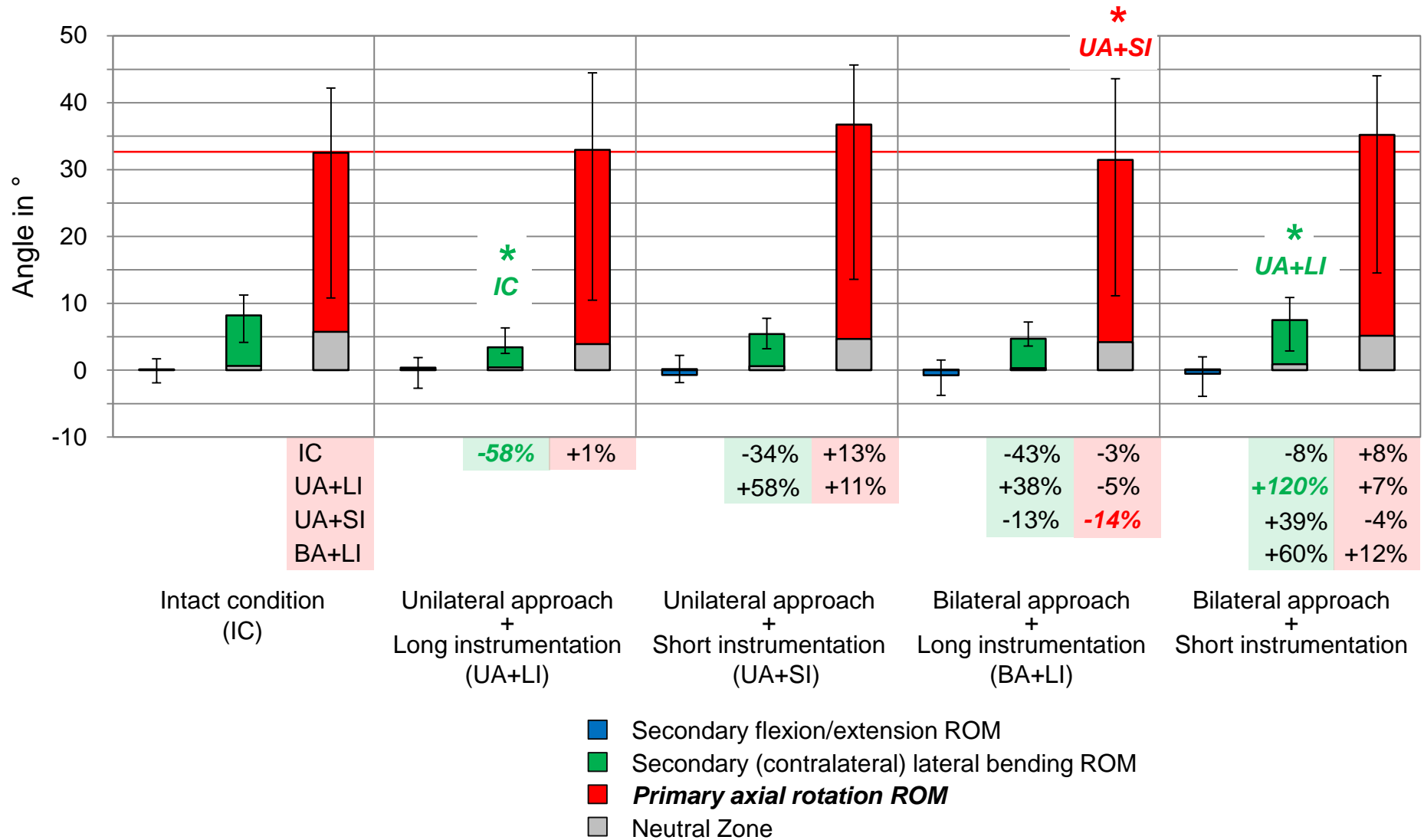

# T3-T4

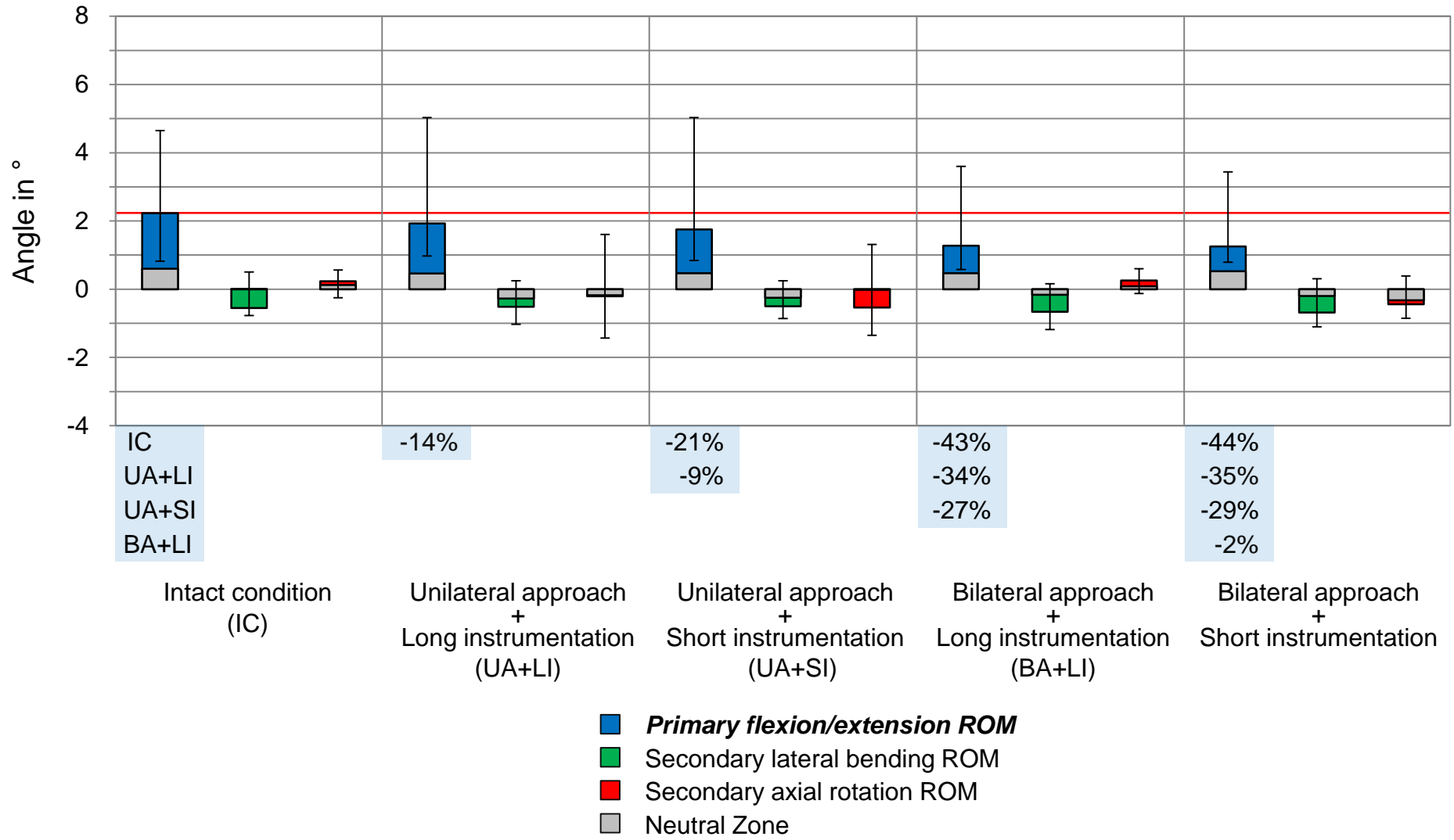

# T3-T4

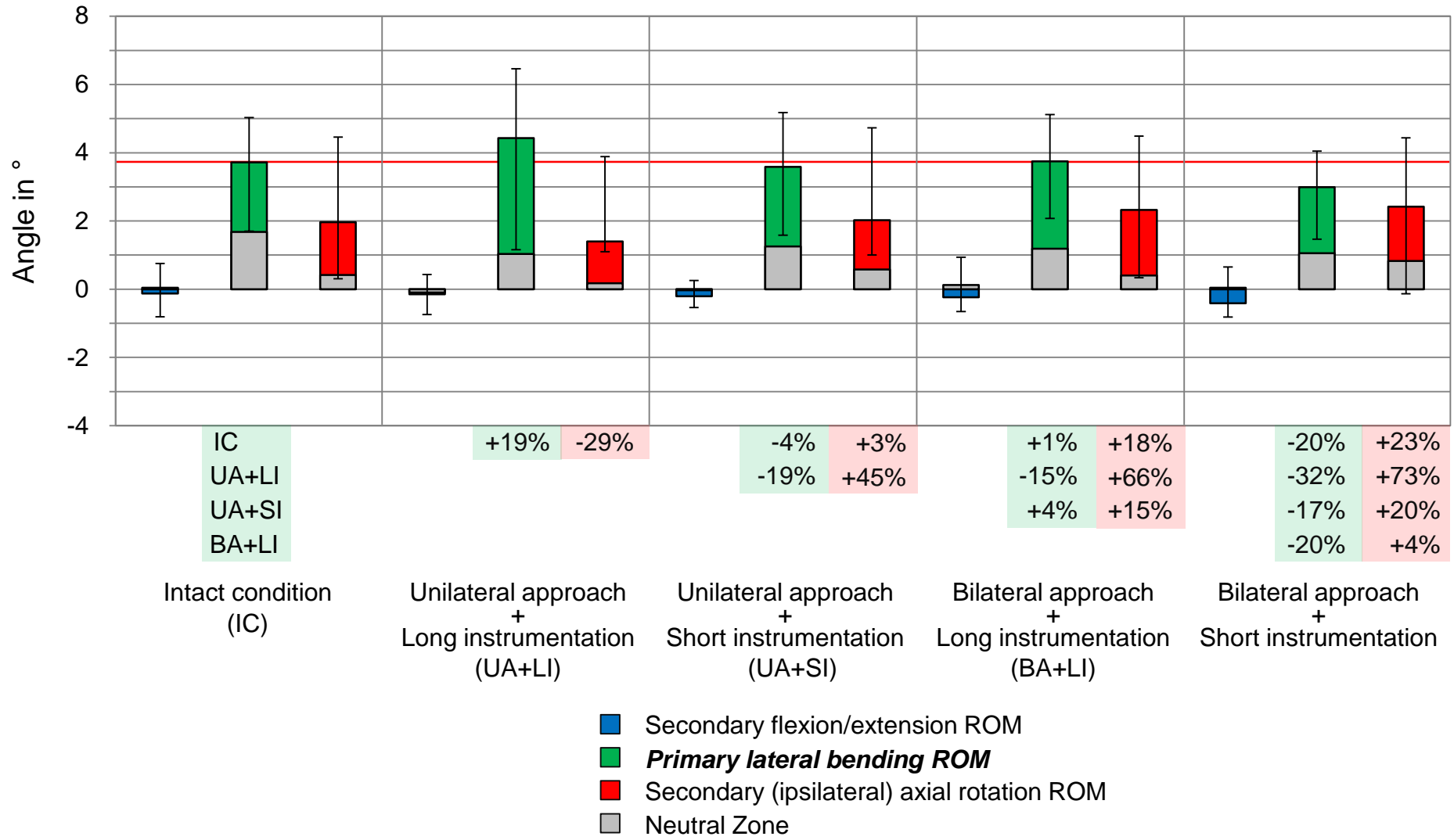

# T3-T4

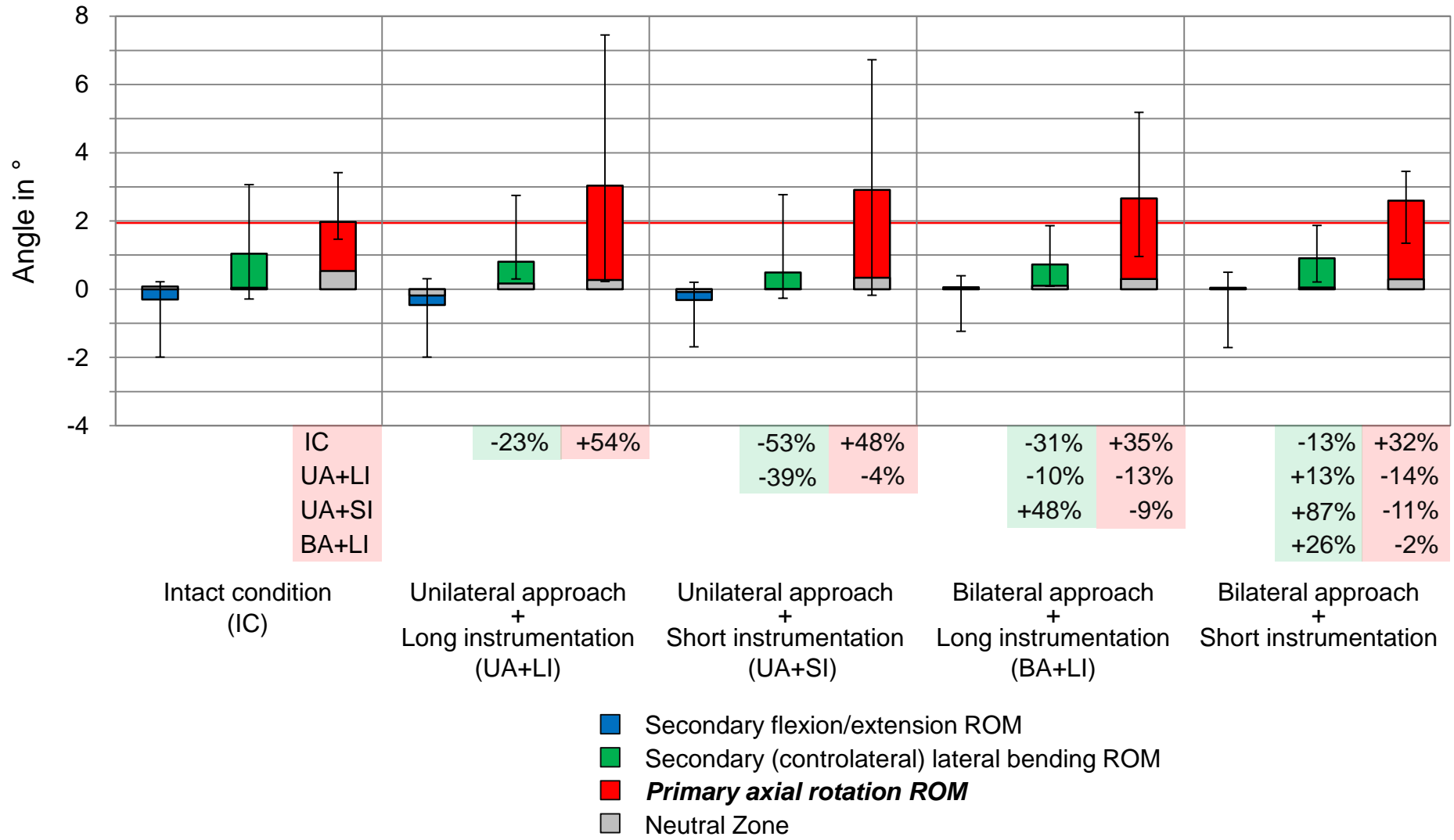

# T4-T5

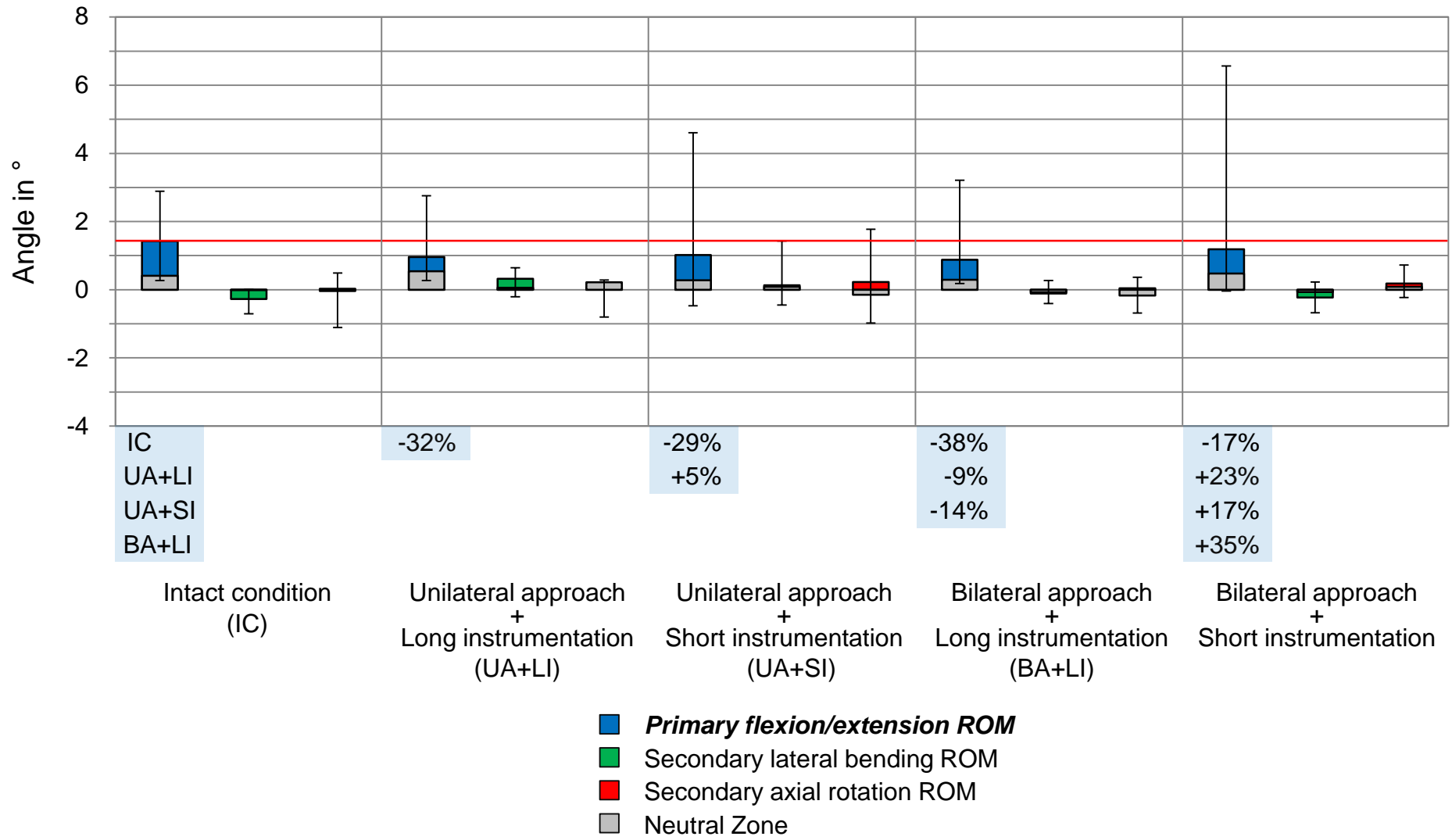

# T4-T5

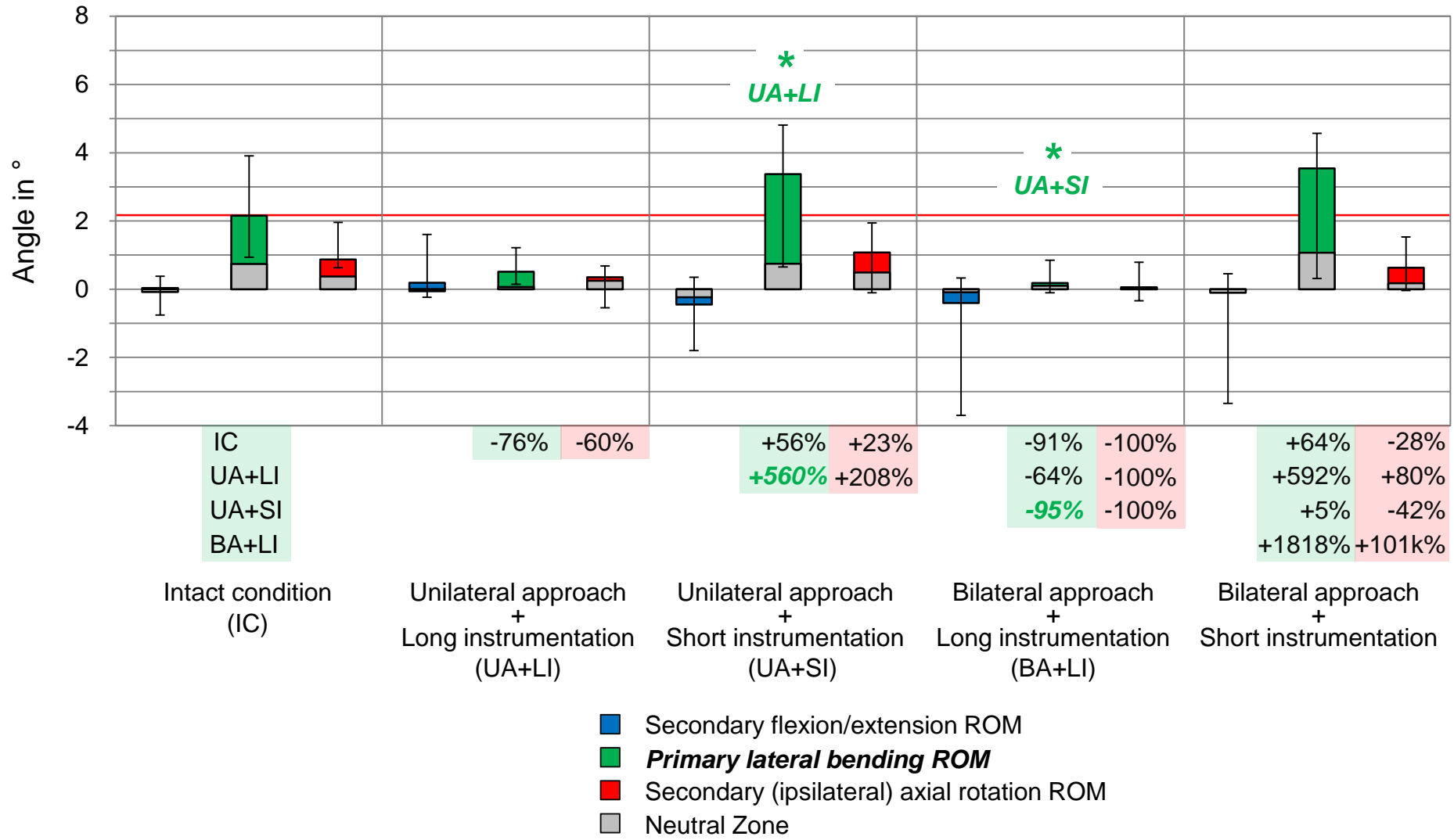

# T4-T5

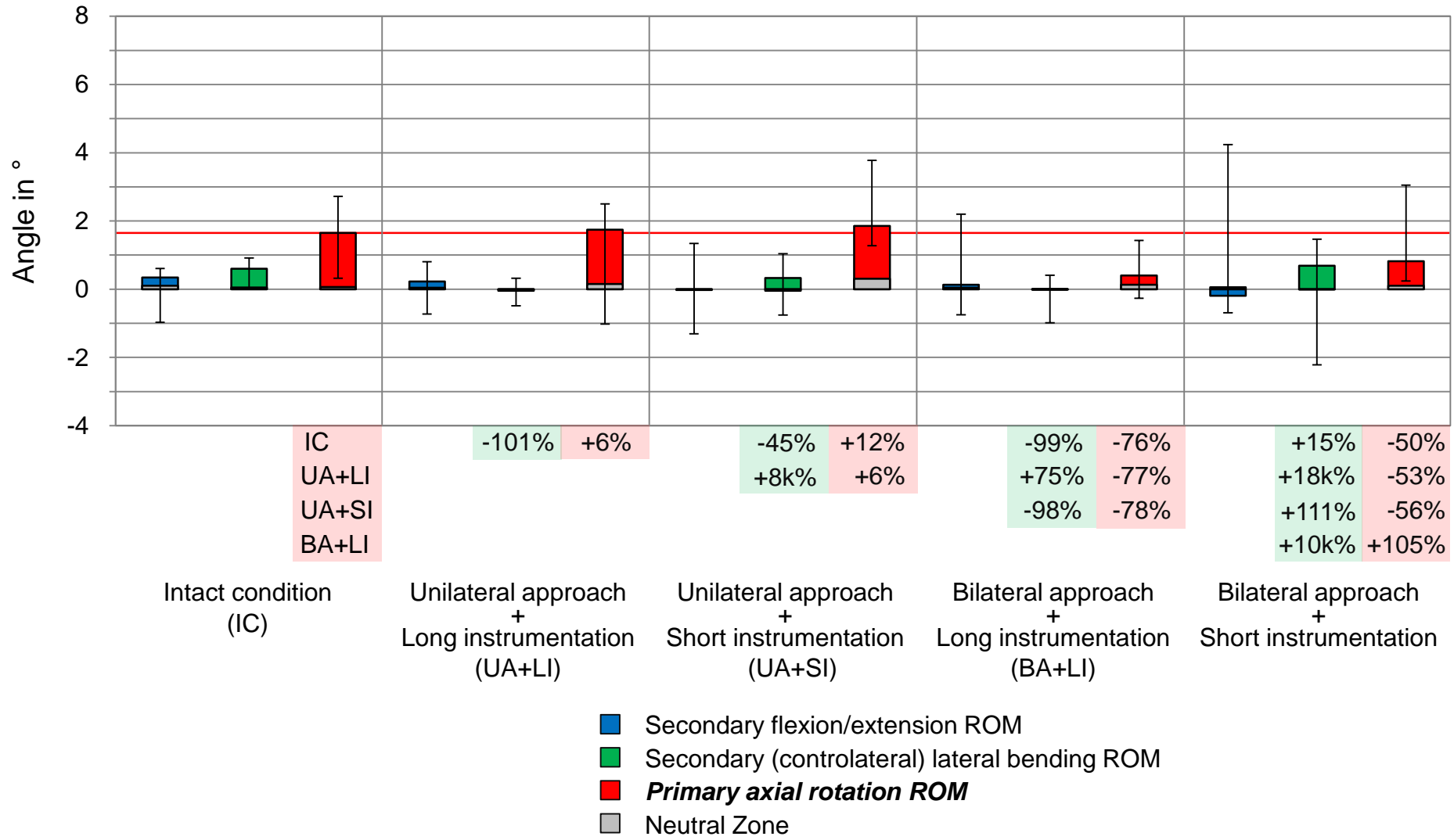

# T7-T8

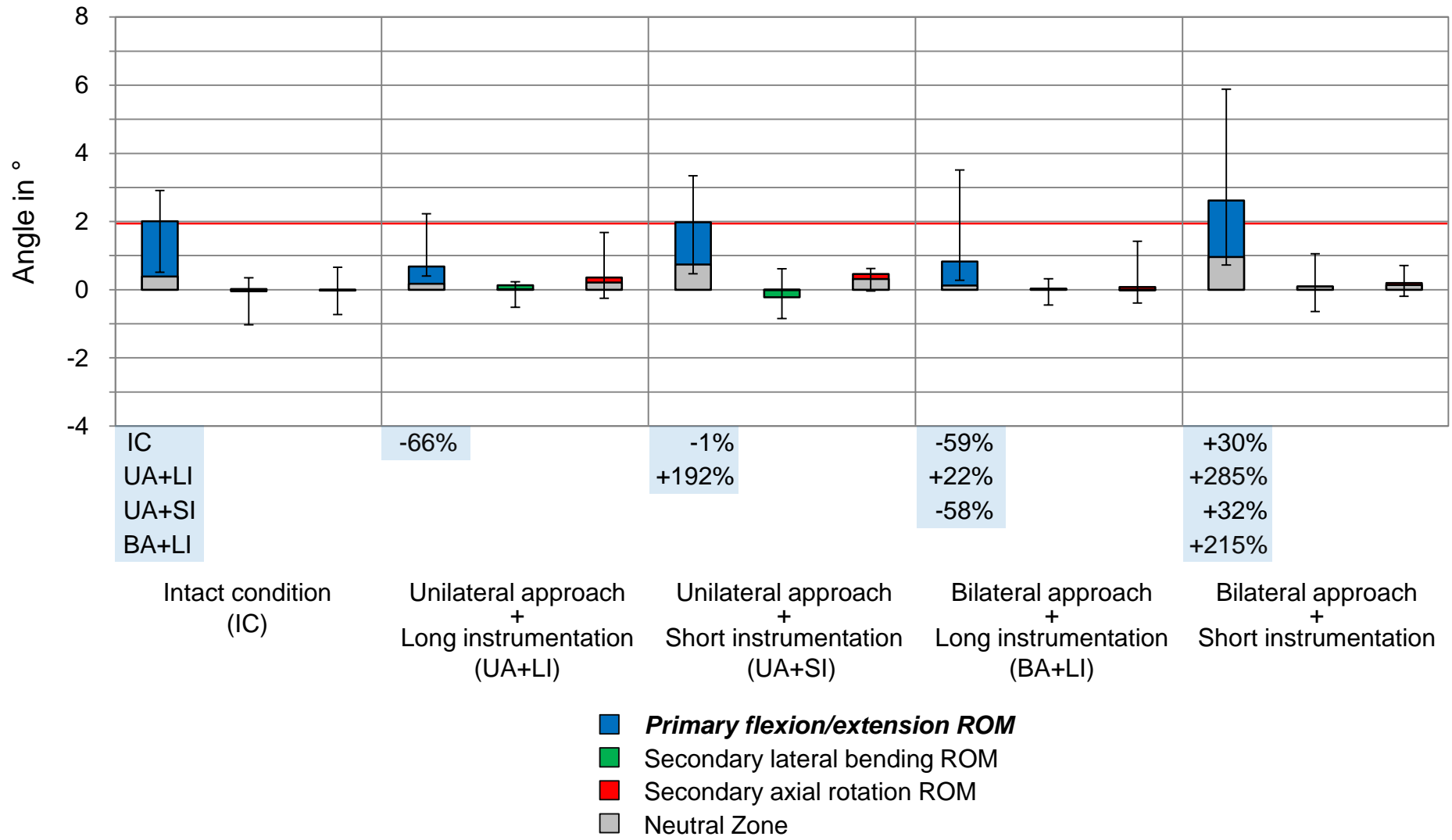

# T7-T8

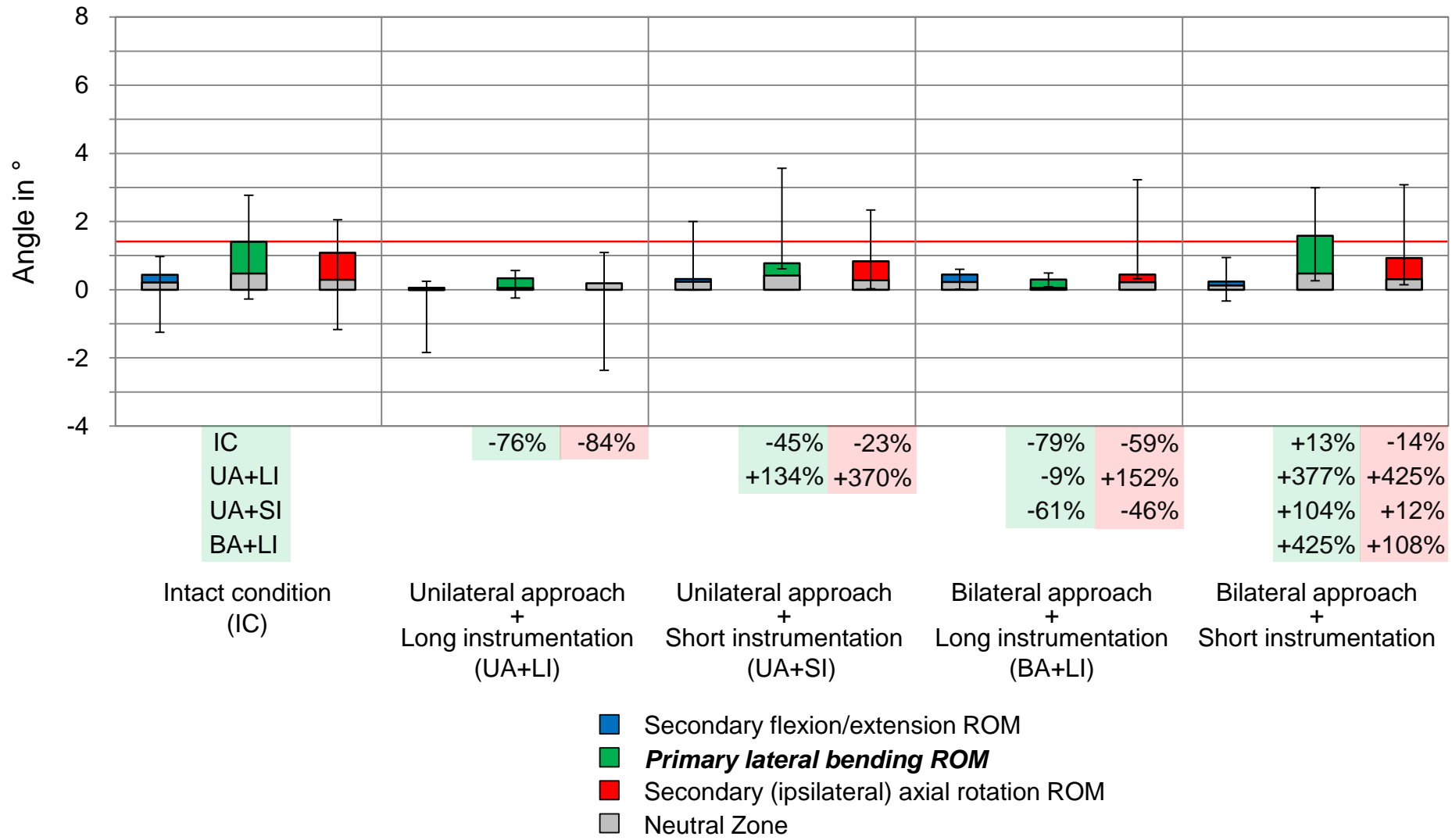

# T7-T8

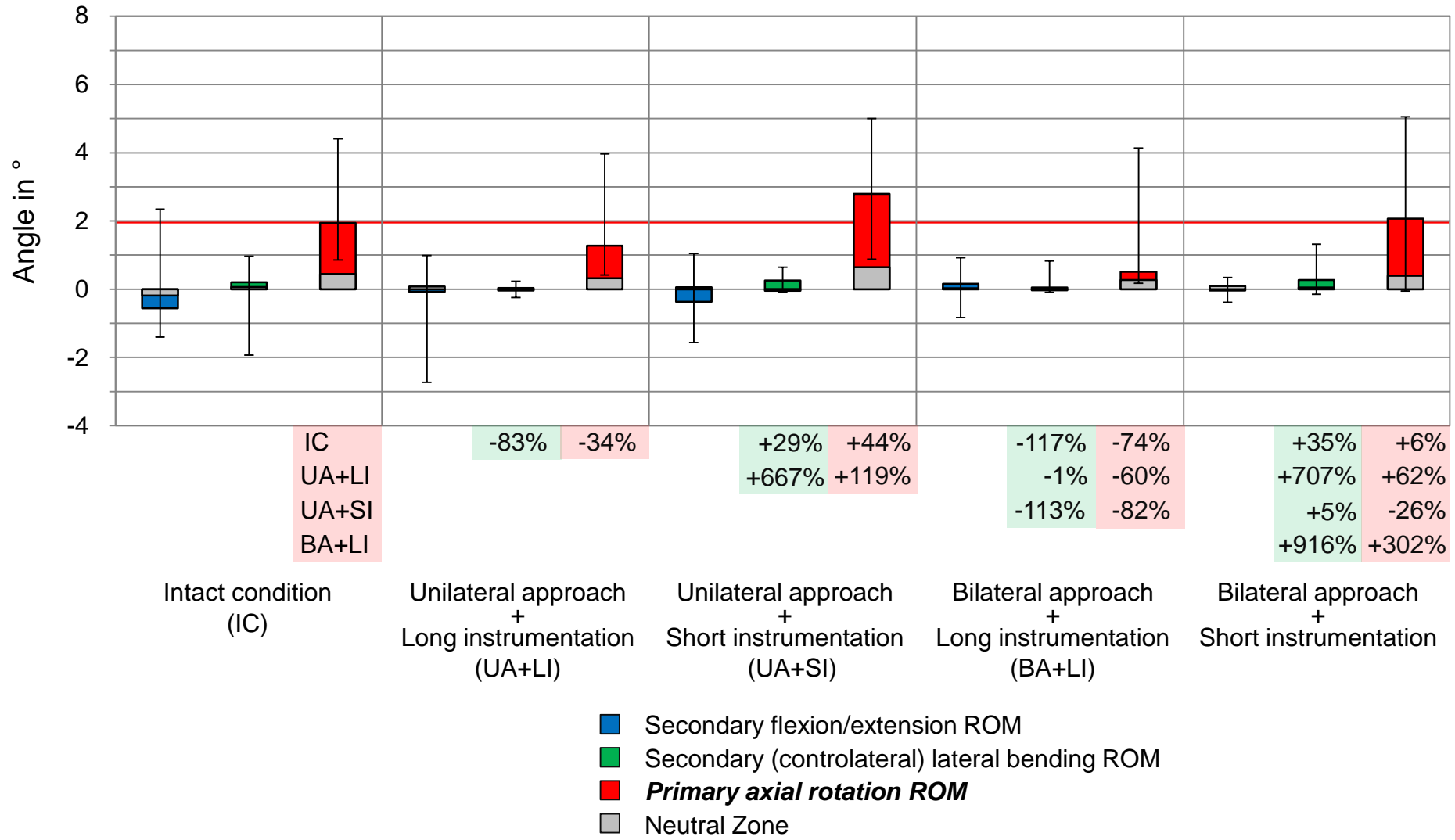

# T8-T9

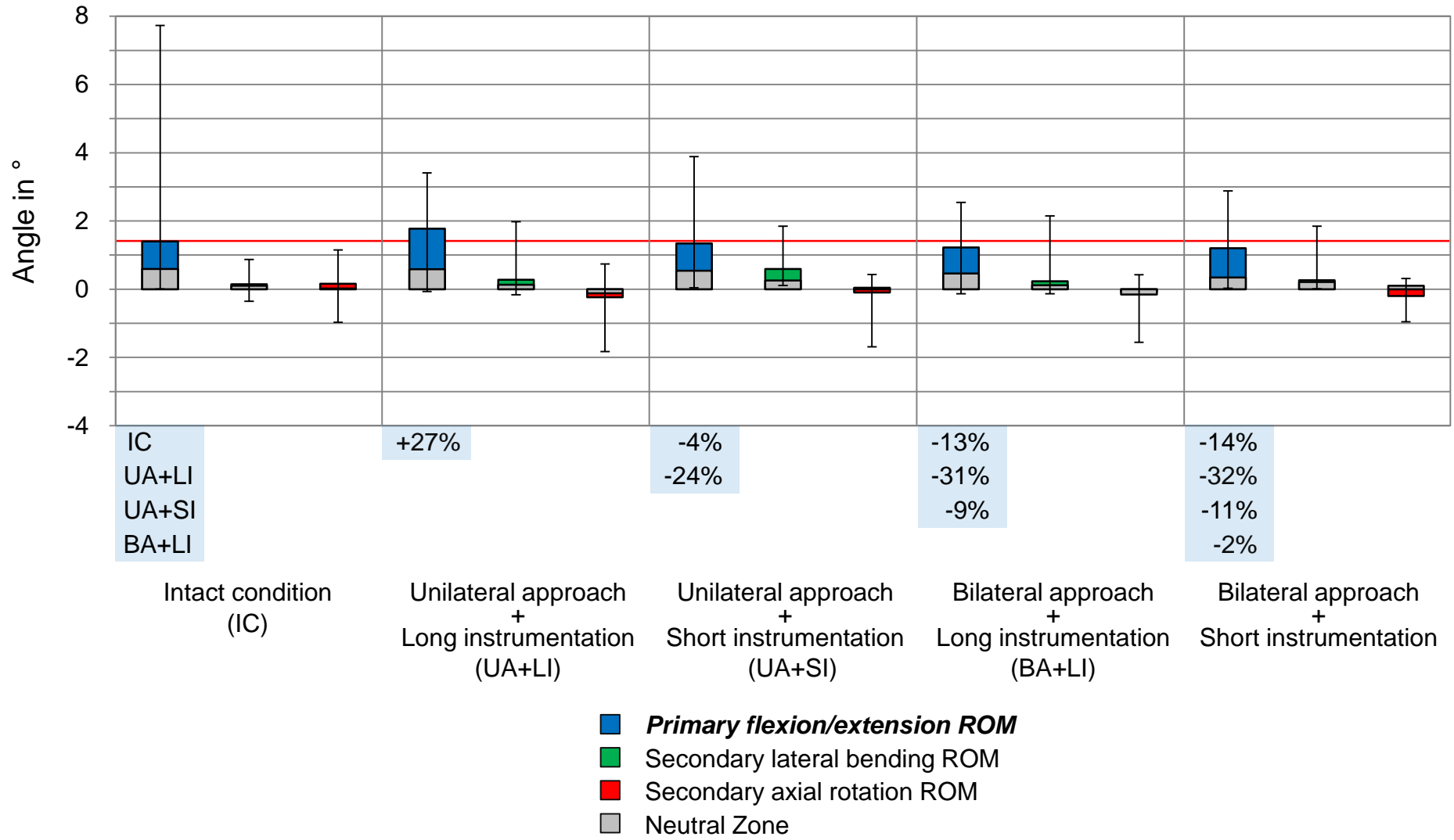

# T8-T9

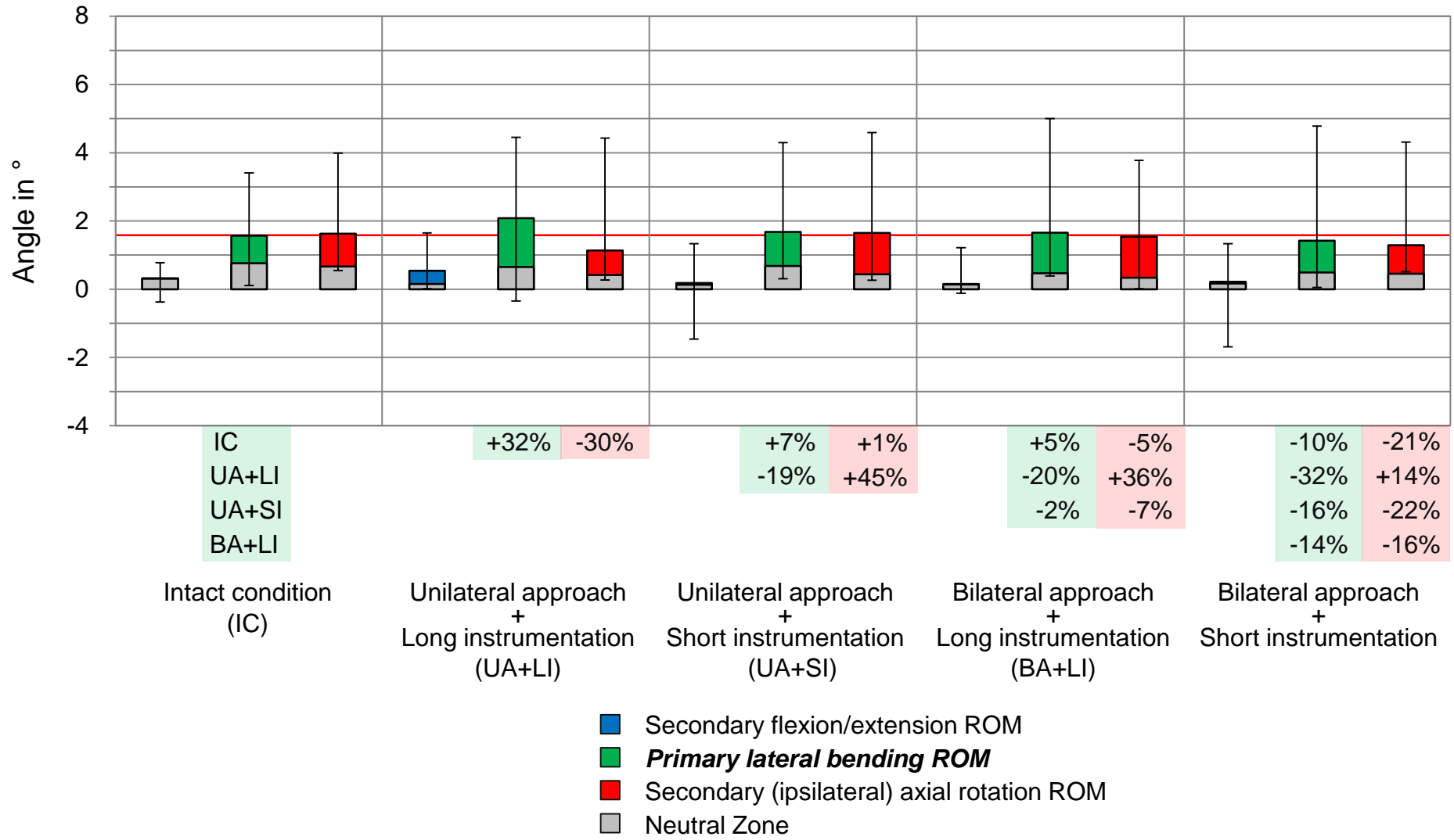

# T8-T9

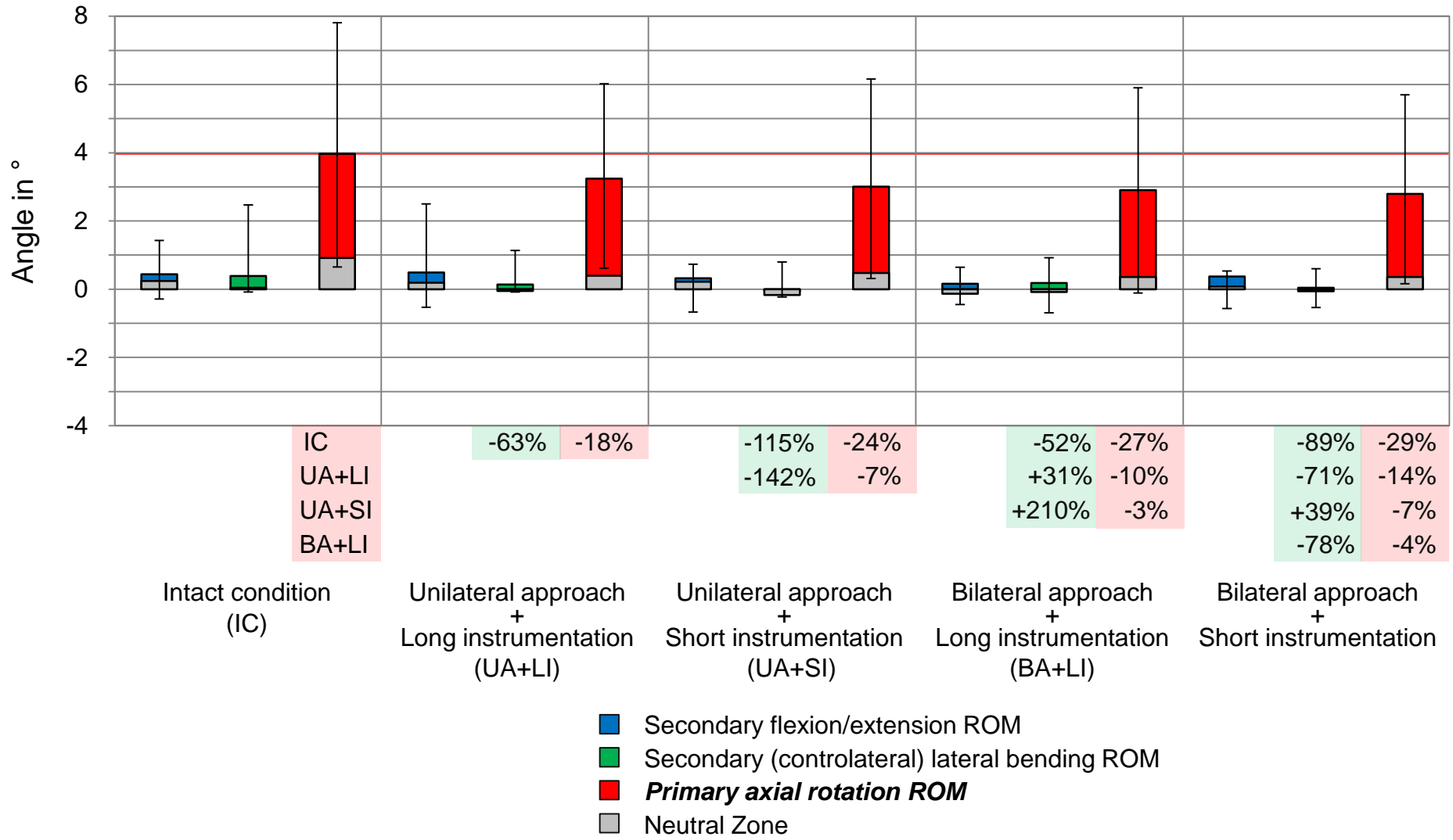

Supplement: Supplementary file 1 [file Data_Sheet_1.PDF]
